# Design and proof of concept of a double-panel TOF-PET system

**DOI:** 10.1186/s40658-024-00674-8

**Published:** 2024-08-23

**Authors:** Andrea Gonzalez-Montoro, Noriel Pavón, Julio Barberá, Neus Cuarella, Antonio J. González, Santiago Jiménez-Serrano, Alejandro Lucero, Laura Moliner, David Sánchez, Koldo Vidal, José M. Benlloch

**Affiliations:** 1 Centro Mixto CSIC - UPV, Instituto de Instrumentación Para Imagen Molecular, Camino de Vera S/N, 46022 Valencia, Spain; 2 Oncovision, C/Jerónimo de Monsoriu, 92 Bajo, Valencia, Spain

**Keywords:** Positron emission tomography (PET), Portable PET, Organ, Specific PET, Double, Panel PET, Time of flight (TOF)

## Abstract

**Objective:**

Positron Emission Tomography (PET) is a well-known imaging technology for the diagnosis, treatment, and monitoring of several diseases. Most PET scanners use a Ring-Shaped Detector Configuration (RSDC), which helps obtain homogeneous image quality but are restricted to an invariable Field-of-View (FOV), scarce spatial resolution, and low sensitivity. Alternatively, few PET systems use Open Detector Configurations (ODC) to permit an accessible FOV adaptable to different target sizes, thus optimizing sensitivity. Yet, to compensate the lack of angular coverage in ODC-PET, developing a detector with high-timing performance is mandatory to enable Time-of-Flight (TOF) techniques during reconstruction.

The main goal of this work is to provide a proof of concept PET scanner appropriate for constructing the new generation of ODC-PET suitable for biopsy guidance and clinical intervention during acquisition. The designed detector has to be compact and robust, and its requirements in terms of performance are spatial and time resolutions < 2 mm and < 200 ps, respectively.

**Methods:**

The present work includes a simulation study of an ODC-PET based on 2-panels with variable distance. The image quality (IQ) and Derenzo phantoms have been simulated and evaluated. The phantom simulations have also been performed using a ring-shaped PET for comparison purposes of the ODC approach with conventional systems. Then, an experimental evaluation of a prototype detector that has been designed following the simulation results is presented. This study focused on tuning the ASIC parameters and evaluating the scintillator surface treatment (ESR and TiO_2_), and configuration that yields the best Coincidence Time Resolution (CTR). Moreover, the scalability of the prototype to a module of 64 × 64mm^2^ and its preliminary evaluation regarding pixel identification are provided.

**Results:**

The simulation results reported sensitivity (%) values at the center of the FOV of 1.96, 1.63, and 1.18 for panel distances of 200, 250, and 300 mm, respectively. The IQ reconstructed image reported good uniformity (87%) and optimal CRC values, and the Derenzo phantom reconstruction suggests a system resolution of 1.6–2 mm.

The experimental results demonstrate that using TiO_2_ coating yielded better detector performance than ESR. Acquired data was filtered by applying an energy window of ± 30% at the photopeak level. After filtering, best CTR of 230 ± 2 ps was achieved for an 8 × 8 LYSO pixel block with 2 × 2 × 12mm^3^ each. The detector performance remained constant after scaling-up the prototype to a module of 64 $\times$ 64mm^2^, and the flood map demonstrates the module’s capabilities to distinguish the small pixels; thus, a spatial resolution < 2 mm (pixel size) is achieved.

**Conclusions:**

The simulated results of this biplanar scanner show high performance in terms of image quality and sensitivity. These results are comparable to state-of-the-art PET technology and, demonstrate that including TOF information minimizes the image artifacts due to the lack of angular projections. The experimental results concluded that using TiO_2_ coating provide the best performance. The results suggest that this scanner may be suitable for organ study, breast, prostate, or cardiac applications, with good uniformity and CRC.

## Introduction

Positron Emission Tomography (PET) is a well-established molecular imaging technology used daily for the diagnosis, treatment, and monitoring of several diseases [1, 2]. All clinical PET scanners use a Ring-Shaped Detector Configuration (RSDC) to take advantage of the angular coverage and symmetries emerging from this geometry, which reduces the complexity of the image reconstruction process and greatly contributes to a better image quality [3]. Yet, conventional scanners, known as Whole-body (WB-) PET, have an axial length between 15–32 cm, thus compromising sensitivity ($\sim$ 1%) and showing spatial resolution values at the center of the Field of View (FOV) in the range of 3–5 mm. This value significantly degrades towards the edges of the FOV [4, 5]. These parameters (in addition to low count rate capabilities, poor reconstructed image corrections (attenuation, scatter…) or, electronical noise, for example) are usually insufficient for certain studies, such as those related to visualizing small lesions in organs like the brain, the prostate, or the heart, among others [6]. To increase sensitivity, it has been proposed to construct large axial coverage scanners, these are the so-called Total Body (TB)-PET. Recently, the EXPLORER system was launched with an axial length of 1.94 m thus covering the entire patient body and reaching 40-fold higher sensitivity than current commercial scanners [7].

Nevertheless, despite the usage of PET is constantly growing, its capabilities for guiding medical interventions such as radiation and hadron therapy, biopsy, and surgery guidance with real-time images are not currently fully covered. Therefore, it is essential to design an imaging system that allows access of the instruments to the organ of the patient during intervention while acquiring data for image reconstruction [8].

To reduce this technological gap, a few different PET systems designs have been proposed using novel and Open Detector Configurations (ODC) with movable parts to enable a flexible FOV that can be adapted to different target areas and patient sizes [8, 9]. However, due to the lack of angular coverage, scanners using ODC configurations provide worse image quality than the conventional ones. In this regard, using Time of Flight (TOF) information could be crucial in reducing image artifacts in open geometries [10] as, for example, the image blur observed in directions coinciding with the missing image spectral cone [11] or, for image attenuation corrections [12].

Recent research has demonstrated that enabling TOF capabilities in PET scanners with ODC designs [13] partially compensates for the lack of information from some directions [14]. TOF information can be used to assign a Gaussian distribution representing the spatial uncertainty along the line of response (LOR). This offers a better estimation of the annihilation position compensating, in part, for the lack of angular information of open geometries, thereby reducing artifacts and improving the reconstructed image quality in terms of uniformity and contrast, thus, enhancing lesion detectability [15, 16]. Note that the width of the assigned Gaussian distribution is proportional to the coincidence time resolution (CTR) of the detectors. The CTR is affected by several factors, namely the intrinsic properties of the scintillators as well as their surface treatment, optical reflectors on scintillation light collection, transit time variations to the photodetector, photodetector technology, crystal-to-photodetector coupling media and configuration, or the electronic readout scheme, among others [17]. Consequently, building high-performance ODC systems requires the development of a detector that is able to provide accurate timing information to boost CTR.

Nevertheless, including TOF is a must to develop a high-quality PET system without giving up the flexibility of an ODC that provides desirable characteristics, like access to the organ during interventions, portability, and application versatility. Indeed, accessing the area during imaging is only possible thanks to the compact design of ODC scanners, which fits the area under exploration and allows the physician to approach (from the lateral and frontal sides) the region of interest without removing the detectors. In the case of conventional PET designs the patient is positioned inside the PET-ring with the area under study (usually) placed at the center of the FOV which is approximately 30 cm apart from the end of the scanner and thus the physician cannot access this region for intervention.

Promisingly, the detector technology has (and stills) improved since the first ODC system appeared. This was in the 1990s, when the group at the University of Geneva (Switzerland) reported on the idea of construction a PET scanner using 40% less detectors than in a full ring scanner. The system was based on two opposite rotating arcs and the detectors used BGO scintillators to allow acquiring full 3D images [18]. The open PET concept was further investigated and, in 2004, a dedicated Prostate PET based on small planar detectors was constructed [19]. Yet, due to the missing angular projections and the lack of TOF the transaxial view of the reconstructed images show artifacts. Only two years later another ODC system, but based on two curved (ellipse: 45 cm minor, 70 cm major axis) movable banks, was launched reporting good phantom images [20]. Then, with the introduction of TOF techniques the interest on ODC PET was reinforced, for example in [21] the authors investigated the feasibility of using dual-headed panel-detectors to build a Region of Interest (ROI)-focused PET scanner, and demonstrated (through simulations) that with enhanced timing resolution, the distortions and artifacts produced by the missing angular information, were reduced.

The open PET concept is also investigated for other applications such as image-guided particle therapy, in situ dose verification and direct tumor tracking [22, 23]. In particular, the use of ODC PET for in-beam monitoring during proton therapy receives great interest. For example, in [24] the authors validated an in-beam PET scanner based on two planar heads of 10 × 25 cm^2^ active area. The PET heads are located above and below the patient, at a relative distance of 60 cm and present a FOV of 25 cm along the beam direction, detector resolution values in the order of the mm were reported. Other applications arising from the ODC PET concept is guiding breast- or prostate-biopsy [25–27]. In [26], the authors presented a design based two asymmetric panels that were constructed using monolithic scintillators, however, since the system lacked of TOF capabilities, the reconstructed images were not accurate enough for biopsy guidance. Similarly, the open concept scanners have been proposed for guiding resection or injection tools (for intra-organ injection) or, to be combined with small insertable PET. For example, in [28] the authors proposed using an internal PET probe for prostate imaging combined with an ODC system to take advantage of the magnification effect; or in [19] where the authors proposed a planar PET system for prostate image and, conclude that, even with limited views, the system enhances the detection of high-uptake lesions.

It has been also proposed constructing ODC scanner for cardiac applications since the open and compact geometry may allow for patient movement and thus for heart-stress conditions without the need of administering drugs [29, 30]. Regarding human-scale ODC PET scanners, an interesting design is reported in [31]. More recently, the walk-through (WT) PET concept was proposed [32], in this design the authors have extended the ODC concept to construct a large axial coverage scanner with a novel, cost-effective, dual flat panel TB-PET system for patients in upright standing positions.

Summarizing, taking advantage of its open design and enhanced spatial resolution (i.e., better lesion localization) [33], different approaches following ODC configurations have already been presented, such as the ones previously mentioned or the ones reported in [8, 13], and references therein.

With the goal of developing a new generation of ODC PET imagers suitable for biopsy guidance and clinical interventions during acquisition, we have been working on a proof of concept (PoC) project to determine the optimal detector characteristics to reach CTR values < 200 ps. Such a CTR value allows constraining the LOR to a segment of 3 cm $(\Delta x$= $c\bullet$ $\Delta t$, where $\Delta x$ is the uncertainty in the LOR, $\Delta t$ can be approximated as the $CTR/2$ and, $c$ is the speed of light in vacuum) [34]; this value should be sufficient to account for the limited angular information and obtain high-quality reconstructed images since the considered distances between panels (200, 250 and 300 mm), which represent the length of the LORs without including TOF information, are larger than 3 cm. Therefore, with 200 ps CTR we will reduce the LORs length within the range of 97% (200 mm panels distance) to 98% (300 mm panels distance) and thus, the image reconstruction process will converge faster and the required information (acquired data) necessary for mitigating the lack of angular information will get reduced. Also, since we are using pixelated-based detectors, retrieving photon depth of interaction (DOI) its complex and, as it has been already stated, in the range of 100–200 ps the contribution of DOI-dependent photon transit times becomes nonnegligible [35] and need to be DOI-corrected. Considering these claims and current limitations in detector technology, targeting for 200 ps is a conservative but, still, good CTR value.

Also, enabling TOF capabilities increases image Signal-to-Noise ratio (SNR) that can be estimated using Eq. (1) [34]. Considering a system with a FOV of 250 mm and the targeted CTR, an SNR boost of ~ $\times$ 2.9 can be expected.

1 $$\frac{{SNR}_{TOF}}{{SNR}_{Non-TOF}}= \sqrt{\frac{2{D}_{eq}}{c\cdot CTR}}$$

where ${D}_{eq}$ is the equivalent object size defined as ${D}_{eq}=D/1.6$ [36].

In particular, we propose a PET system based on two panels in which the distance between panels can be adjusted to maximize sensitivity while allowing guided interventions [37]. To validate the motivation towards the proposed design and justify that this ODC system is capable to perform as a ring-shaped system with all angles covered, section *B.1 Imaging performance: conventional ring-shaped vs the proposed 2-panel ODC PET*, provides a simulation comparison of the Derenzo and Image Quality phantoms of the ODC scanner with the ones simulated in a conventional brain dedicated PET system.

The proposed ODC design faces different technological challenges, such as the optimization of the detector block in which the scintillator selection and its surface treatment and configuration will be a key factor to achieve high CTR performance [17, 38–40], the development of an electronic acquisition system capable of providing the required CTR while reducing the complexity of the scanner [41], and a software reconstruction method adapted to our specific geometry.

The present manuscript describes the proposed ODC PET system geometry and reports our efforts to develop and implement a detector capable of reaching the targeted CTR. In particular, the article provides a simulation study of the expected system performance, including an evaluation, following the NEMA NU-4 2008 protocol [42], of: (i) the expected system sensitivity as a function of different panel distances, (ii) the contrast recovery coefficients (CRC) and image uniformity of the IQ phantom, and (iii) the Valley to Peak ratios of the rods in the Derenzo phantom. The results are provided with and without including TOF information during the image reconstruction to emphasize the need for TOF when dealing with ODC PET systems. Note that, for the evaluation, the NEMA NU-4 2008 (for small animal systems) protocol has been selected instead of the NEMA NU-2 2012 (for conventional human-size scanners) since the size of the proposed scanner is more similar to the ones of preclinical scanners and thus the measurement and phantom specifications of the NEMA NU-4 2008 match better with our design [43].

In the following, experimental results are provided, showing the CTR and photopeak relative gain of a prototype detector. This detector is based on a (LuY)_2_SiO_5_ (LYSO) pixel array coupled to a matrix of 8 × 8 Silicon Photomultipliers (SiPMs), and its dependency with two different crystal surface treatments: Enhanced Specular Reflector (ESR) [44] and TiO_2_ coating [45]. The detectors are connected to a TOFPET2 ASIC [46] from PETsys to ensure scalability. Previous works have also explored the influence of different crystal treatments on CTR [17, 38, 39]. However, to our knowledge, this is the first evaluation performed for a compact PET module in which the TOFPET2 ASIC digitalizes the output signals. Finally, it also reports the assembly of a complete detector module and its performance in pixel identification (i.e., spatial resolution) and CTR.

## Materials and methods

In the following, we report on (A) the proposed ODC-PET system geometry, (B) a simulation study of this system, and (C) the detector module design and its experimental evaluation.

### A. system geometry

Considering the average size of a human torso and based on previous designs [29], the dimensions of the PET panels have been selected to be 256 $\times$ 256 $\times$ 12 mm^3^ each. The distance between the panels is not fixed and can be changed in the 200 to 300 mm range. Each one of the panels consists of a matrix of 4 $\times$ 4 modules, and each module is built using small LYSO scintillator pixels of 2 $\times$ 2 $\times$ 12 mm^3^ each from TACrystal Co., Ltd, Taiwan. The LYSO pixels are arranged in 4 $\times$ 4 matrixes of 8 $\times$ 8 pixel elements each.

**Fig. 1:**
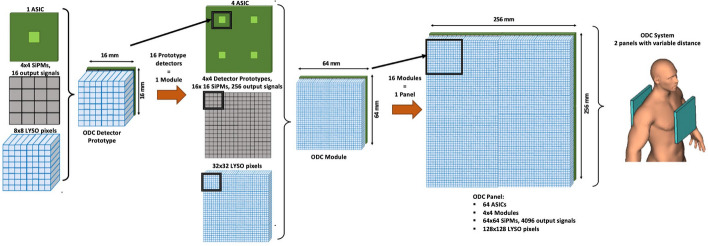
From left to right, prototype detector element and components; detector module and components; and full panel detector. A sketch of the system being used as a torso imager is provided for a visual representation of the system

Each group of 4 $\times$ 4 scintillation matrixes is coupled using optical grease (Saint-Gobain, BC-630 Silicone Grease) [47] to an array of 4 $\times$ 4 AFBR-S4N44P164M BroadCom® SiPMs [48], each with an area of 4 $\times$ 4 mm^2^. The SiPMs arrays are mounted on a Printed Circuit Board (PCB) designed explicitly for the project that allocates 4 $\times$ 4 basic SiPM arrays to form a module of 64 × 64 mm^2^. The scintillator, module, and panel will be assembled identically to match and cover the sensitive surfaces of the SiPM detectors. Figure 1 provides drawings of the proposed system and the main components contained in 1 panel.

Regarding the detector block (scintillators & SiPMs) output signals, these are directly fed to the PETsys TOFPET2 ASIC boards, which are connected to the PETsys FEM acquisition card, and finally, to the PETsys data acquisition system (DAQ) for pre-processing. A total of 16 modules of 64 × 64 mm^2^ each, will be combined in a 4 × 4 matrix to form one panel of 256 × 256 mm^2^. Thus, a total of 32 modules are required to construct the two-panel system.

In the next stage of the project, it is planned to include a custom analog electronic readout between the SIPMs and the ASIC. The multiplexing readout is based on a previous design [49] and provides a reduction topology to shrink the number of signals to be digitalized in a ratio of 4 to 1. This is accomplished by merging the analog signals from the SiPMs allocated in the same row or column [41, 50]. Note that, after summation of the signals, there will be a common anode for all SiPMs belonging to one row (or column) thus allowing to preserve the rise time slope, which is key to achieve a significant reduction in crosstalk between the temporal channels of adjacent pixels and, thus, reduce the parasitic capacity of the connected SiPMs. Based on previous results with a semi-monolithic detector, see [49], we do not expect significant degradations in CTR performance. Finally, the multiplexed signals are connected to the PETsys chain.

Table 1 reports the number of elements needed to build the proposed ODC system with and without including the reduction readout topology.

**Table 1:** Number of elements needed to build the proposed ODC system with and without including the reduction readout topology

	PET Module		PET system	
	All signals	Reduction readout	All signals	Reduction Readout
Panel	–	–	2	2
Module	1	1	2 $\times$ 16 = 32	2 $\times$ 16 = 32
ASIC	4	1	2 $\times$ 64 = 128	2 $\times$ 16 = 32
Channels	256	64	2 $\times$ 4096 = 8192	2 $\times$ 1024 = 2048
*FEM_128_	2	1	64	16
*FEM_256_	1	½	32	8

*The number of FEMs will depend on the final design and component availability. Either the 128 or the 256-channel FEMs will be used

### B. system simulations

**Fig. 2:**
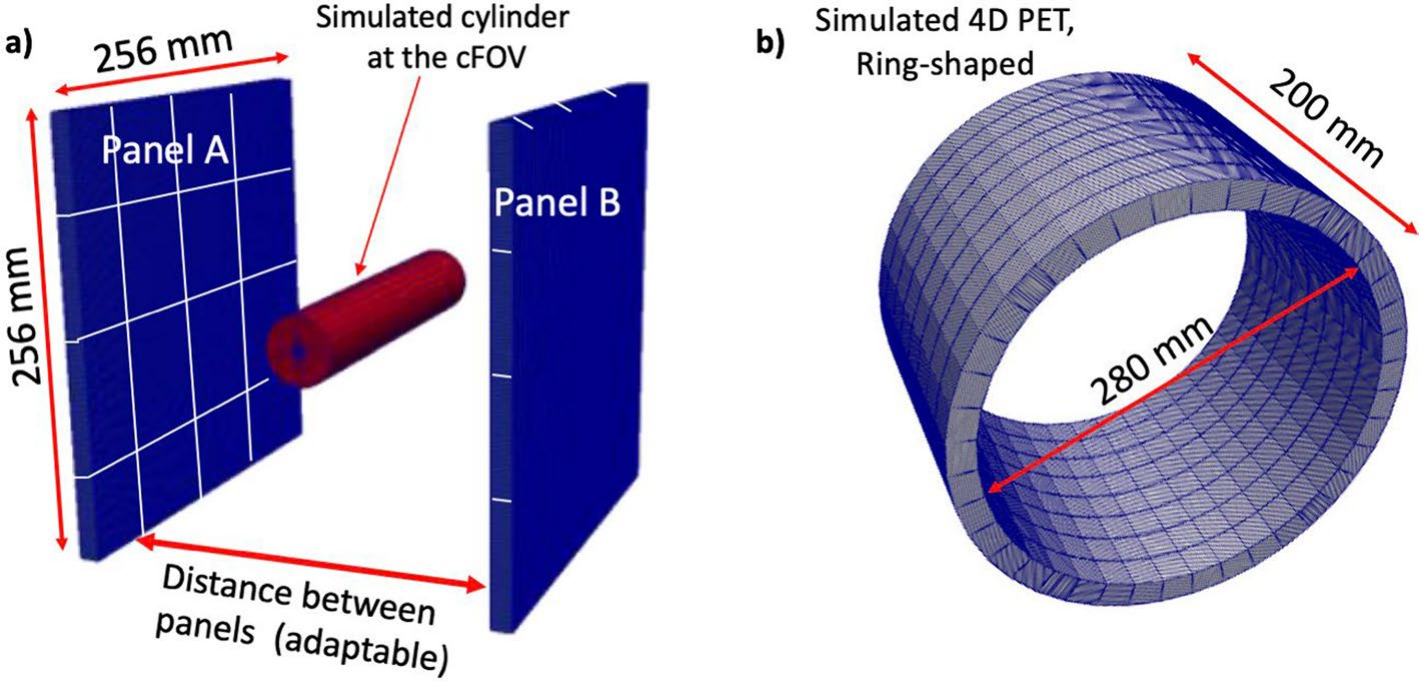
**a** Simulated geometry for the proposed ODC PET system based on two panels with variable distance. The simulated cylinder is also depicted. **b** Simulated geometry of the 4D PET, see [63]

To evaluate the expected performance of the double-panel design, simulations were performed using GATE v9.2 [51, 52]. The mechanical design of the system was based on previous studies [53, 54] aiming at ODC scanners. In this particular case, the aperture between panels can be adapted for different distance values to provide enough space for diverse patient sizes. In particular, the system has been simulated with panel distances of 200, 250, and 300 mm; these values have been selected since they are optimal for pediatric, standard-size, and plus-size patients, respectively. Figure 2a shows the simulated system with a phantom cylinder. This phantom is a solid cylinder composed of high-density polyethylene (0.95 g/cm^3^), the dimensions are 25 mm in diameter and 256 mm long.

In all simulations, the physics list “emstandard_opt4” was used, which is the list recommended for medical applications [55, 56] and, an energy blurring of 11% was included in the GATE code. Regarding the digitizer settings, the simulations include: TOF = 180 ps with an energy window of 357—613kev (30% below the photopeak for scatter correction and, 20% above the photopeak), and a coincidence windows of 5 ns. The simulations incorporate the main detector experimental components namely, optical coupling between the SiPMs and the Scintillation pixels (refractive index, n = 1.46), the treatment of the scintillator walls (polished surfaces + reflector/coating), air gap between elements.

For image reconstruction, a Maximum Likelihood Expectation Maximization (MLEM) algorithm was implemented [57], and an energy window in the range of 357 to 613 keV has been used. Detailed information regarding the MLEM software framework can be found in [58]. Also, TOF capabilities have been implemented in the reconstruction algorithm using the differences of timestamps of the coincidence events and binning the coincidence and scatter events in different histograms for each time bin [59]. During each MLEM iteration, a forward and backward projection for all the time bins was performed, weighting the system matrix considering the time boundaries in each bin [60]. Finally, scatter and attenuation correction have also been implemented in the reconstruction process to improve image quality [61]. For scatter correction, a double-energy window method was applied as detailed in [62], the events falling within the scatter window (± 30% at the photopeak level) were used to estimate the correction factor which is later used during the reconstruction. Regarding the attenuation correction, synthetic (simulated) μ-maps have been generated with the same dimensions and positions as all simulated phantoms and then, included in the MLEM reconstruction platform.

The sensitivity of the system has been calculated following the NEMA 2008 protocol [42] for the three mentioned panel distances (200, 250, and 300 mm).

Then, a study to find the number of time bins that maximize image quality was performed using a simulated image quality (IQ) NEMA NU-2 2008 phantom [42]. The NEMA IQ phantom has 50 mm length and 30 mm in diameter. It has two regions namely, rod-area (hot) and uniform-area that cover 20 mm and 30 mm axially, respectively. The rod-area contains cylinders of 1, 2, 3, 4, 5 mm in diameter and 20 mm in length. The uniform-area has two cold regions (filled with air and water) that occupy half of the axial plane (15 mm) and are of 8 mm in diameter. Thus, the total volume of the phantom is 35.3 cm^3^ and the active one is 35.32—V_cold_ = 35.2–1.5 = 33.8 cm^3^. In this simulation, the IQ phantom background was filled with a 5.3 kBq/ml activity and the hot rods with a 4:1 ratio. The IQ image was reconstructed with 20 iterations and a voxel size of 1 mm. A CTR value of 180 ps was selected since the targeted timing resolution for the system is < 200 ps. In particular, this selection was motivated by the fact that when we started the simulations, we only had data of two single collimated pixel elements with TiO_2_ coating. This pixel-to-pixel set-up yielded CTR values in the range of 170–187 ps FWHM depending on the acquisition conditions, and we felt confident at some point we will be reaching this value (after scaling up to a detector element and then to a module). Nevertheless, the Derenzo phantom analysis (explained in the following) was repeated but considering 200 ps CTR and no significant differences were observed in resolution.

The final reconstructed image of the phantom was also used to estimate the image Uniformity (see Eq. 2) and the Contrast Recovery Coefficients (CRC) (see Eq. 3).

2 $$Uniformity=100 \times \left(1-\frac{{STD}_{roi}}{{Mean}_{roi}}\right)$$

3 $$CRC=100 \times \left(\frac{{A}_{m, ROI}-{A}_{m, background}}{{A}_{t, ROI}-{A}_{t, background}}\right)$$

For the analysis, the image slices covering the central 10 mm length of the IQ rods were averaged to obtain a single image slice of lower noise, and circular ROIs were drawn around each rod with a diameter that was twice the physical diameter of the rods. The maximum values in these ROIs were measured and calculated for both the non-TOF case and the TOF using 3, 5, 7, 9, and 11 time bins.

A simulation of a Derenzo phantom with rod diameters of 1.0, 1.2, 1.6, 2.4, 3.2, and 4.0 mm and an injected activity of 10 MBq was performed to assess image resolution. The simulation consisted on a 30 min long PET data acquisition and was reconstructed with 20 iterations and a 0.5 mm voxel size.

The reconstructed image was used to evaluate the accuracy in resolving rods in the Derenzo phantom using the Valley-to-Peak ratios (see Eq. 4). The Rayleigh criterion was applied to estimate the resolvability and, thus, the image spatial resolution of the proposed scanner; see equivalence 5 and reference [64].

4 $$\%Valley\;to\;Peak= 100 \times \left(\frac{Avg.\;Valley\;Voxel\;Values}{Avg.\;Peak\;Voxel\;Values}\right)$$

5 $$Rayleigh\;Criterion=0.735 (73.5\%)>Valley\;to\;Peak$$

### B.1 imaging performance: conventional ring-shaped vs the proposed 2-panel ODC PET

The IQ and Derenzo phantom simulations detailed in *Section. B* have been repeated but using a conventional ring-PET. These results are compared with the ones reported by the 2-panel PET to verify the claim that including TOF information during the PET image reconstruction process partially compensates for the lack of angular projections and thus, systems like the proposed 2-panel PET can perform similarly to conventional ring-shaped PET in which all angular views are covered.

In particular, simulations of a brain-dedicated PET system have been used. The system is the so-called 4D PET, which consists on a conventional cylindrical scanner with a total of 320 semi-monolithic detectors arranged in 8 rings, see Figure 2b. The system defines an axial length of 200 mm and an internal diameter of 280 mm, and has 3D photon positioning and TOF capabilities. For specific details on the 4D-PET technology see reference [63].

Regarding image reconstruction, the same MLEM algorithm and the same settings than the ones used for the 2-panel ODC simulations have been implemented in the 4D PET case. Both non-TOF and TOF reconstructions are provided to validate the claim that including TOF capabilities during image reconstruction compensates for the missing angular projections. In the TOF reconstructions, CTR values (determined through the experimental evaluation of the modules [63]) of 350 ps and 180 ps FWHM have been used for the 4D PET and the ODC system, respectively. For the comparison, slices of the coronal and axial views of the phantoms, the source projection profiles of the smallest rods in the Derenzo phantoms of both systems, and the CRC values for the IQ phantom rods (non-TOF and ToF cases), are provided.

### C. the prototype: detector element and module

Following the specifications described in the previous sections, a prototype detector element consisting of an 8 $\times$ 8 matrix of LYSO pixels with sizes of 2 $\times$ 2 $\times$ 12 mm^3^ each, coupled to a matrix of 2 $\times$ 2 AFBR-S4N44P164M BroadCom® SiPMs, was built.

Since the crystal surface finish and treatment play an important role regarding the transmission of the scintillation light to the photosensor and thus impacts CTR, spatial, and energy resolutions, two different crystal treatments were studied:

i. All pixel elements have polished surfaces and are covered (except one of the 2 $\times$ 2 mm^2^ faces, which is in contact with the photosensor) with an Enhanced Specular Reflective (ESR) foil. ESR are high reflectivity, mirror-like optical films. The selected ESR for the experiments has a reflectance of about 98.5% (VikuitiTM ESR film (3M, USA)) [43].
ii. All pixel elements have polished surfaces and are coated (except the face in contact with the photosensor) with TiO_2_ white coat. The TiO_2_ is a type of coat composed of a titanium dioxide pigment mixed with water (soluble coat) [45], which acts as a diffuse reflector [65] and presents enhanced reflectivity for longer wavelength scintillators such as LYSO.

**Fig. 3:**
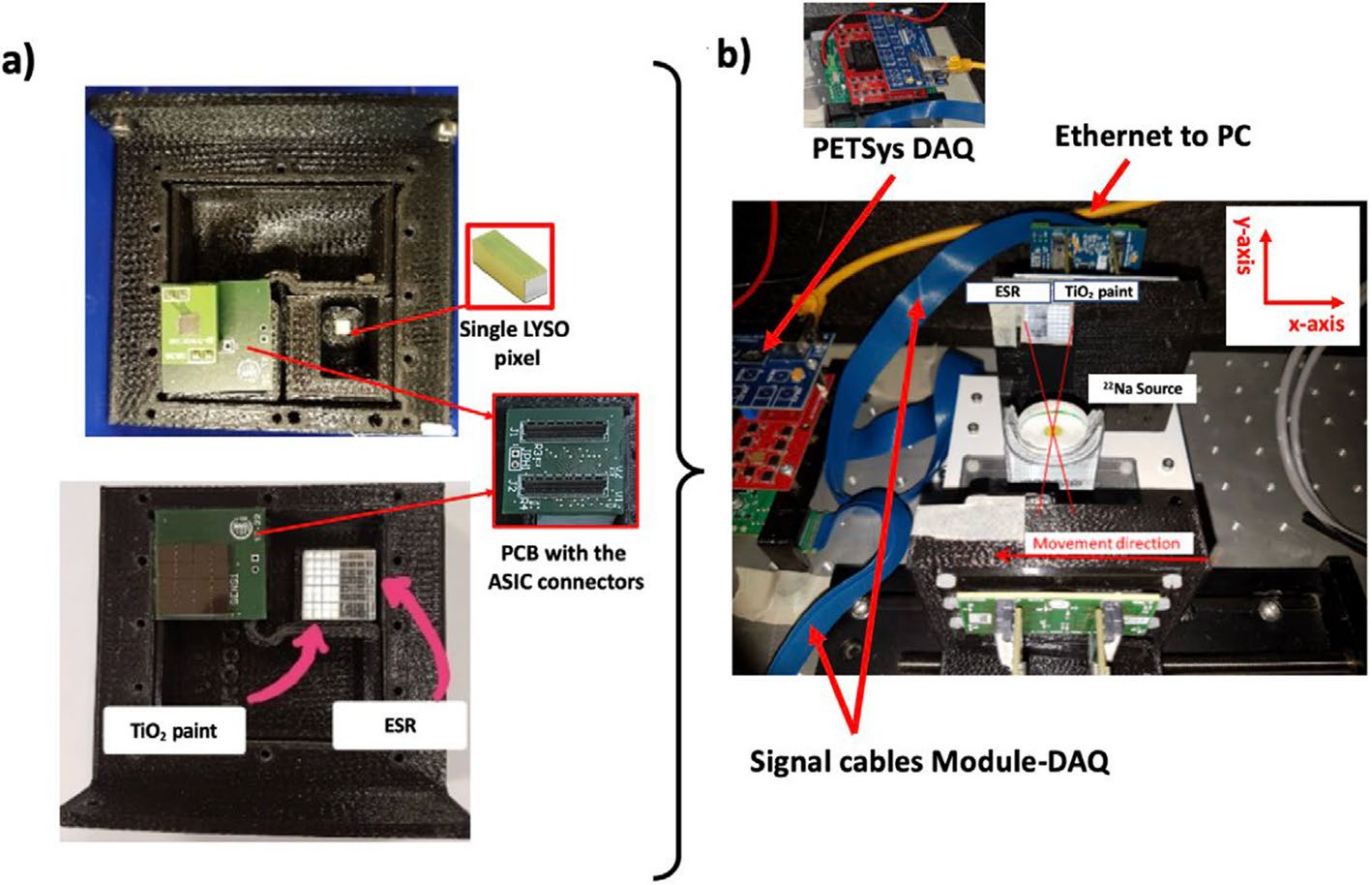
Actual photos of reference and evaluation prototype detector. As can be seen in panel **a** the scintillation matrix includes the two treatments (TiO_2_ and ESR). **b** Photos of the experimental setup for coincidence measurements are shown indicating a label with the x- and y-axis orientations

For the experimental evaluation, the setup consisted of one of the described detector prototypes but in which the 4 $\times$ 8 crystals on the left side are covered with ESR, and the 4 $\times$ 8 crystals on the right side are coated with TiO_2_, see Fig. 3. The SiPM outputs were connected to the PETsys DAQ and then sent to the workstation for analysis. A Python code was implemented for this purpose.

To acquire coincide data, a reference detector based on a single 2 $\times$ 2 $\times$ 12 mm^3^ LYSO pixel coupled to a single SiPM was used. Both the evaluation and reference detectors were placed inside a black box to shield the modules from ambient light and were cooled down using compressed air. The detector (including the electronic board) temperature was kept in the range of 22 $\pm$ 1 °C for all acquisitions.

Coincidence data was acquired by placing a ^22^Na source between the detectors; see the photos in panel (b) of Fig. 3. In the setup, the detector under evaluation was fixed on an XY table, and the reference one & source were sequentially moved simultaneously to evaluate both treatments.

Different combinations of these parameters were tested to determine the best PETSys threshold configuration [46] and best SiPM overvoltage (SiPM_OV_). In particular, the following values were considered: SiPM_OV_: [5, 9, 13], Th_E_: [15], Th_1_: [5, 11, 17, 23] and Th_2_: [5, 11, 17, 23]. A total of 48 combinations (for each treatment) were evaluated (see Table 2). Each acquisition lasted 20 min and was repeated three times to account for possible variances.

**Table 2:** SiPM_OV_ and PETsys Threshold configuration combinations

			SiPM	PETsys parameters		
#Measurement	#Number in plot (ESR)	#Number in plot (TiO_2_)	Over voltage (OV)	Th_E_	Th_1_	Th_2_
1	14	43	5	15	5	5
2	31	46	5	15	5	11
3	33	20	5	15	5	17
4	20	41	5	15	5	23
5	18	45	5	15	11	5
6	29	13	5	15	11	11
7	26	44	5	15	11	17
8	17	27	5	15	11	23
9	19	48	5	15	17	5
10	39	31	5	15	17	11
11	37	14	5	15	17	17
12	3	42	5	15	17	23
13	11	39	5	15	23	5
14	1	24	5	15	23	11
15	30	1	5	15	23	17
16	25	40	5	15	23	23
17	21	7	9	15	5	5
18	9	2	9	15	5	11
19	42	33	9	15	5	17
20	48	5	9	15	5	23
21	36	19	9	15	11	5
22	4	21	9	15	11	11
23	5	6	9	15	11	17
24	43	47	9	15	11	23
25	22	37	9	15	17	5
26	6	23	9	15	17	11
27	41	16	9	15	17	17
28	35	29	9	15	17	23
29	24	38	9	15	23	5
30	47	15	9	15	23	11
31	46	10	9	15	23	17
32	23	26	9	15	23	23
33	15	22	13	15	5	5
34	7	11	13	15	5	11
35	45	36	13	15	5	17
36	38	35	13	15	5	23
37	40	34	13	15	11	5
38	10	30	13	15	11	11
39	16	32	13	15	11	17
40	32	28	13	15	11	23
41	28	9	13	15	17	5
42	12	25	13	15	17	11
43	44	8	13	15	17	17
44	34	3	13	15	17	23
45	13	18	13	15	23	5
46	2	17	13	15	23	11
47	8	4	13	15	23	17
48	27	12	13	15	23	23

A total of 48 measurements were performed for each crystal treatment.

These measurements were used to estimate the CTR and the relative photopeak gain, which is a good estimation of the light transfer to the photosensor and, thus, of the expected energy performance. Both the CTR and photopeak gain have been estimated as the Full-Width-At-Half-Maximum (FWHM) of the coincidence time difference between the evaluation and reference detectors and the energy spectra, respectively. The experimental errors have been calculated as the standard error calculated for three measurement trials and for each combination of parameters.

Once the surface treatment and the acquisition parameters that yield better performance were determined, two full modules, 64 $\times$ 64 mm^2^ each, were mounted. Each component was carefully studied and mounted using custom-made holders for the scalability process.

**Fig. 4:**
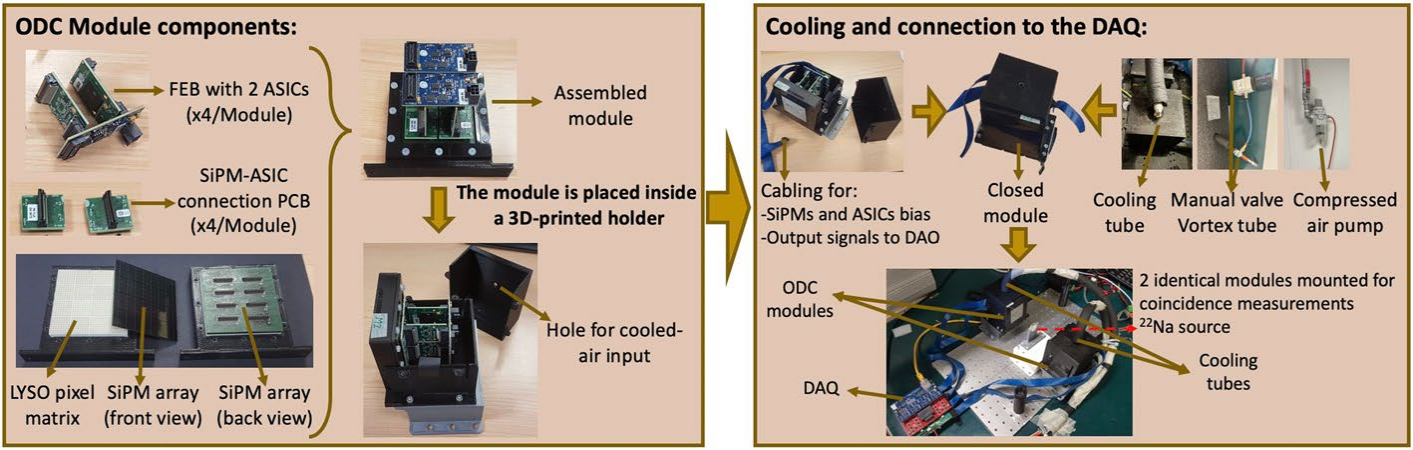
Photos showing the scaling-up process of the prototype detector to a full module. As depicted, the module comprises 16 scintillation blocks (64 LYSO pixels each) with TiO_2_ coat as treatment, 64 SiPMs, and 4 ASICs. A holder and black box were designed and 3D printed to ensure light shielding and to keep the temperature constant (using compressed air). Finally, a photo of the experimental setup for module evaluation (including PETsys DAQ) is shown

Each module was built using four previous prototype detectors, i.e., 4 $\times$ 4 matrixes of 8 $\times$ 8 pixel elements each. All pixel elements were treated using TiO_2_ coating (see Section Results. B). Figure 4 shows the scaling-up process and the components used for constructing the modules.

The two identical modules were placed inside a dark box, cooled down, and used to acquire coincidence data. The same non-collimated ^22^Na source was placed closer to one module between the detectors to increase the solid angle coverage and obtain a more homogeneous irradiation of one of the detectors. The Flood map was used to estimate the pixel resolvability and, thus, the expected spatial resolution. For CTR calculations, ROIs were drawn around clusters of 2 $\times$ 2 scintillation pixels (1 SiPM, 4 $\times$ 4 mm^2^) at five different random positions, namely: 1 $\times$ corner (SiPM (1,1)), 2 $\times$ lateral (SiPMs (2,6) and (14,14)), and 2 $\times$ center (SiPMs (8,8), (10,9)). The acquired data was corrected for time-skew and, the coincidence events were energy filtered, only those coincidences falling within a ± 30% of the photopeak value were considered for CTR calculations. Then, the coincidence photon arrival time were histogram for each ROI and fitted using a Gaussian distribution, the total (module) CTR was finally estimated as the mean value of the CTRs for each ROI.

## Results

### A. simulation results

**Fig. 5:**
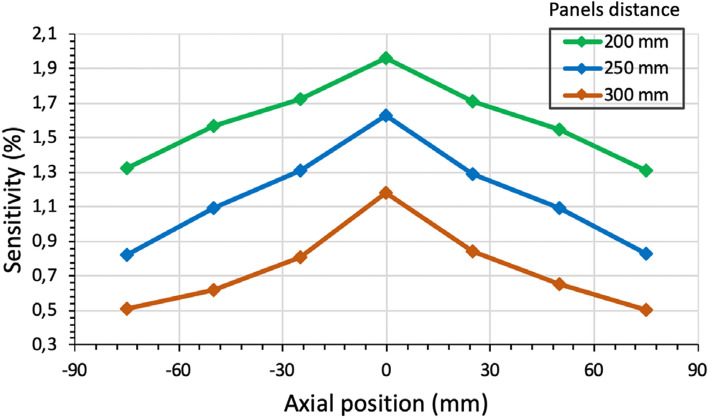
Simulation results for the system sensitivity at different axial positions for three different panel distances

Figure 5 reports the estimated sensitivity at different positions along the transaxial, between panels, direction of the scanner for each one of the considered panel distances. Table 3 shows the sensitivity values obtained at the center and edge of the FOV for each case. Note that the center of the FOV is defined as axial position = 0 mm (i.e., middle point between the two panels) and, edges of the FOV is defined as axial positions =  ± 85 mm.

**Table 3:** Sensitivity values at the center (axial position = 0 mm) and edges (axial position =  ± 85 mm) of the ODC system FOV for the three simulated panel distances

Distance between panels (mm)	Sensitivity (%)	
	FOV center	FOV edge
200	1.96	1.31
250	1.63	0.83
300	1.18	0.50

**Fig. 6:**
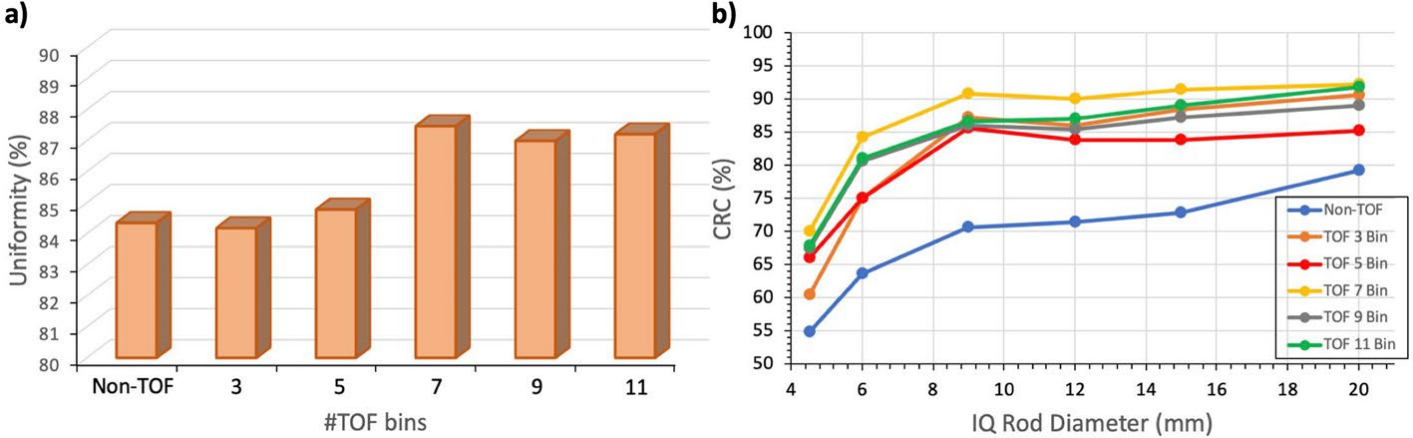
**a** Percentage Uniformity values obtained for the IQ phantom and for each different number of TOF bins. **b** CRC values as a function of the number of TOF bins for each one of the IQ phantom rods

The histogram and plot shown in Fig. 6a and b, report the uniformity and CRC calculated values for the IQ reconstructed image, respectively, using 20 iterations and a voxel size of 1 mm. These values are presented as a function of the number of TOF bins used in the simulation. The best results are obtained when using seven time bins. Using fewer bins reduces the uniformity and CRC values, while using more bins requires additional computational time and memory resources with minimal improvements in image quality. Yet, there is a number of TOF bins (n’) at which the image starts to converge and thus, considering a number of bins beyond that one (n’) does not improves the reconstructed image. Indeed, the deviation values observed in the Uniformity values between the 7 and 9 bins cases, and between the 9 and 11 bins cases, are of 0.5% and − 0.25%, respectively, thus these variations are minimal. Consequently, for the rest of the reconstructed images, seven time bins were used to maximize the performance of the reconstruction algorithm.

Note that, the reported uniformity values for the non-TOF and 3 TOF bins cases are 84,35% and 84,14%, respectively. Although comparable, the non-TOF case is slightly better than the 3 TOF bins one, this is because when using 3 TOF bins the reconstructed FOV is divided in three histogram regions which, for the studied system, is not sufficient to provide an improvement in the image quality when compare with the one histogram case (non-TOF).

For the abovementioned configuration of seven TOF bins, an image uniformity of 87.5% was obtained and, regarding CRC, best values of 70.2, 84.4, 90.8, 90.0, 91.5, and 92.3 (%) were obtained for the 4.5, 6, 9, 12, 15 and 20 mm rods, respectively.

**Fig. 7:**
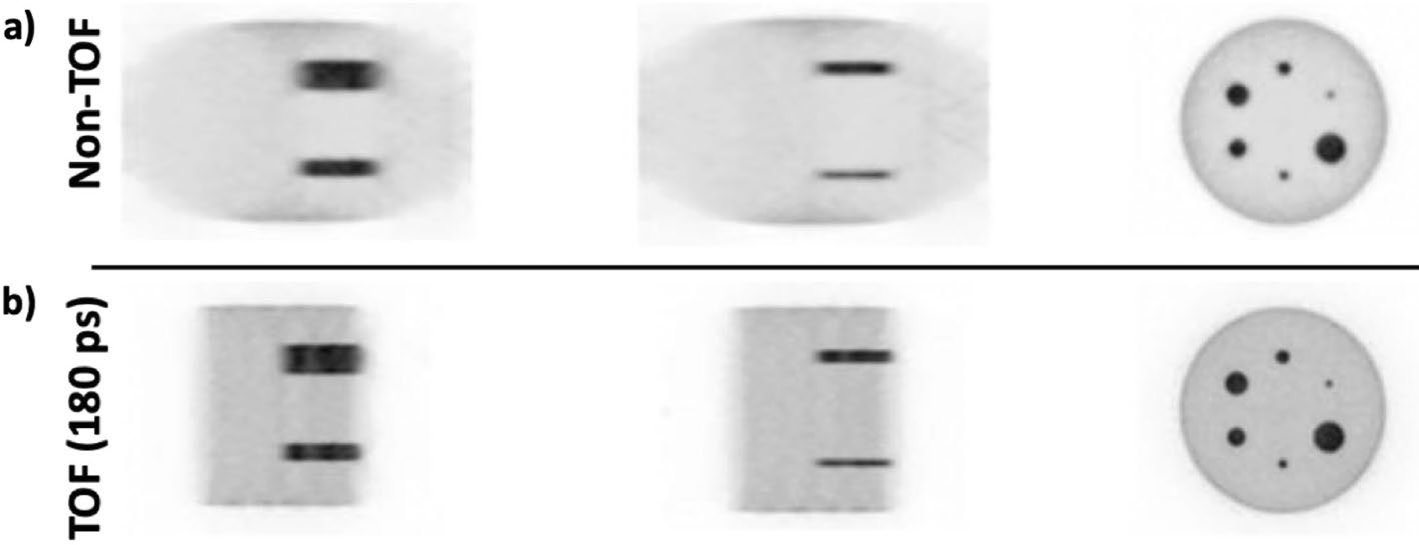
Reconstructed images of the IQ phantom without **a** and with **b**, enabling TOF capabilities in the reconstruction software

Figure 7a and b, show the reconstructed IQ phantom images without and with TOF information (180 ps) during reconstruction, respectively. The reconstructed images shown in Fig. 7 has been obtained considering the best settings based on the conclusions extracted from the simulated data shown in Fig. 6b. In particular, these images were reconstructed with 20 iterations and a voxel size of 1 mm, and, as can be seen, all 6 rods are clearly resolved in both the non-TOF and TOF cases. However, the image quality and contrast are notably better in the TOF case. Moreover, in the axial slices, the non-TOF case exhibits deformation at the edges due to the lack of angular coverage. In contrast, for the TOF case the distortion is completely compensated and the IQ phantom is properly visualized.

**Fig. 8:**
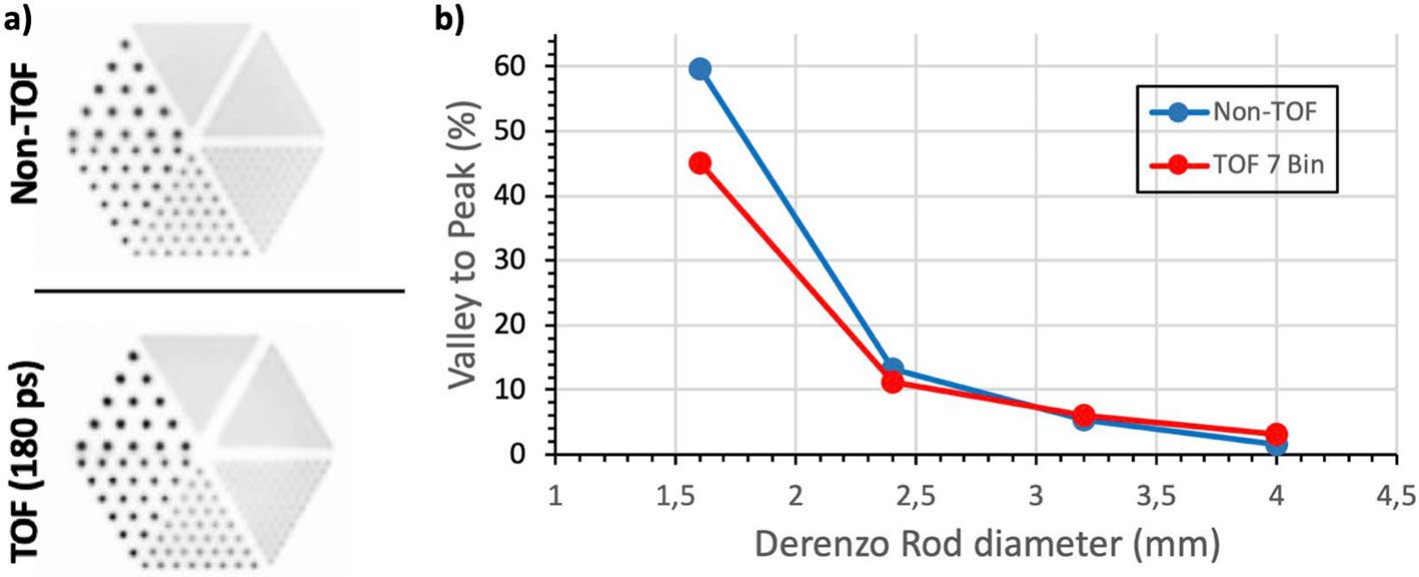
**a** Reconstructed images of the Derenzo phantom without (top) and with (bottom), enabling TOF capabilities in the reconstruction software. The 1.6, 2.4, 3.2, and 4 mm rods are distinguished. **b** Valley to peak ratios calculated for the 1.6, 2.4, 3.2, and 4 mm rods in the Derenzo phantom image

For the Derenzo phantom, the image reconstruction was performed with 20 iterations and a voxel size of 0.5 mm. Figure 8, (a-top), and (a-bottom), shows the Derenzo reconstructed image without and with including TOF information (180 ps, 7 TOF bins), respectively. As in the IQ phantom case, better image quality and contrast were obtained for the TOF case. As can be seen, the two smallest sets of rods (1.0 and 1.2 mm) are not resolved, but the rest (1.6, 2.4, 3.2, and 4 mm) are distinguished.

The plot in Fig. 8b, reports the calculated Valley to Peak ratios for each resolved rod diameter (1.6, 2.4, 3.2, and 4 mm) and for the non-TOF and TOF cases. As expected, better ratios are obtained for the TOF case, being the impact of including TOF more noticeable for the smaller rods. In all cases, the Valley to Peak ratios are below 73.5% (Rayleigh criterion^64^); thus, the expected reconstructed image resolution is within the 1.6–2 mm range.

### A.1 imaging performance, conventional ring-shaped vs the proposed 2-panel ODC PET

**Fig. 9:**
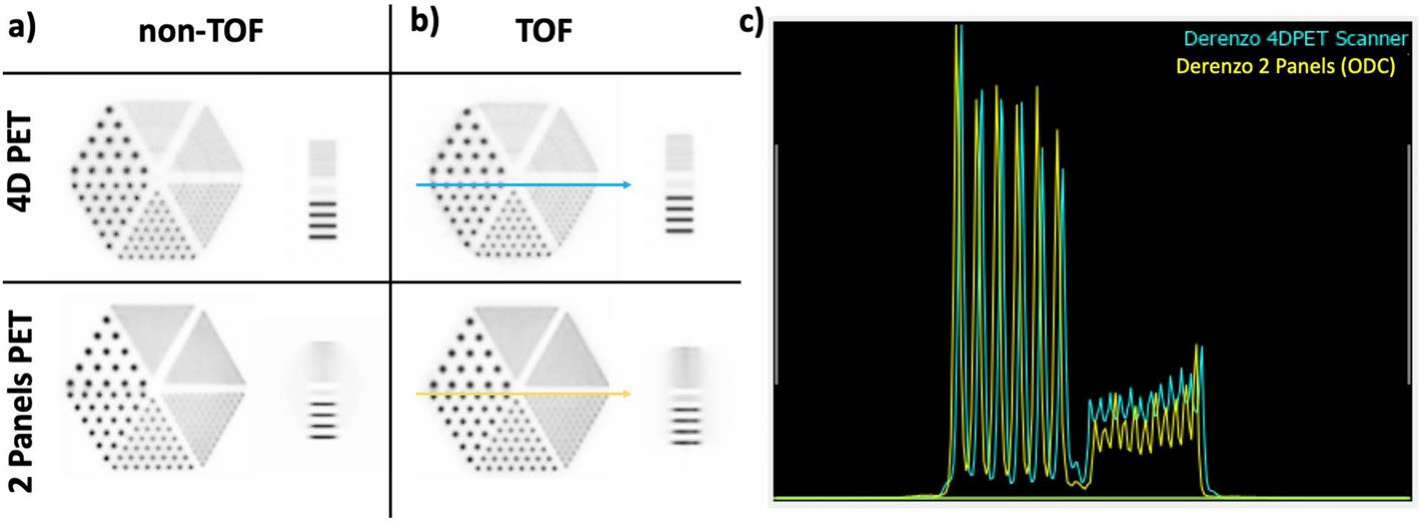
**a** and **b**, reconstructed images of the Derenzo phantom without and with enabling TOF capabilities in the 4D PET (top) and ODC system (bottom) simulations, respectively. **c** projection profiles of the 3.2 mm and 1.6 mm rods marked with the yellow and blue arrows in panel (**b**)

Figure 9a and b show the coronal and axial slices of the reconstructed Derenzo phantom simulations for the 4D PET (top) and the 2-panel ODC system (bottom), for the non-TOF and TOF cases, respectively. As can be seen, the effect of including TOF information impacts more the ODC images since the axial view improves and the halo observed in the 2-panels non-TOF case starts to vanish as the lack of angular data gets compensated.

Figure 9c shows the projection profiles of the Derenzo 3.2 mm and 1.6 mm rods (marked with blue and yellow arrows in panel (b), for the 4D PET and the 2-panel ODC, respectively). As depicted, the rod profiles are comparable thus demonstrating the capabilities of the system to perform as a conventional ring-shaped scanner when TOF is included but, with less components thus, reduced mechanical, electronical and investment complexities.

**Fig. 10:**
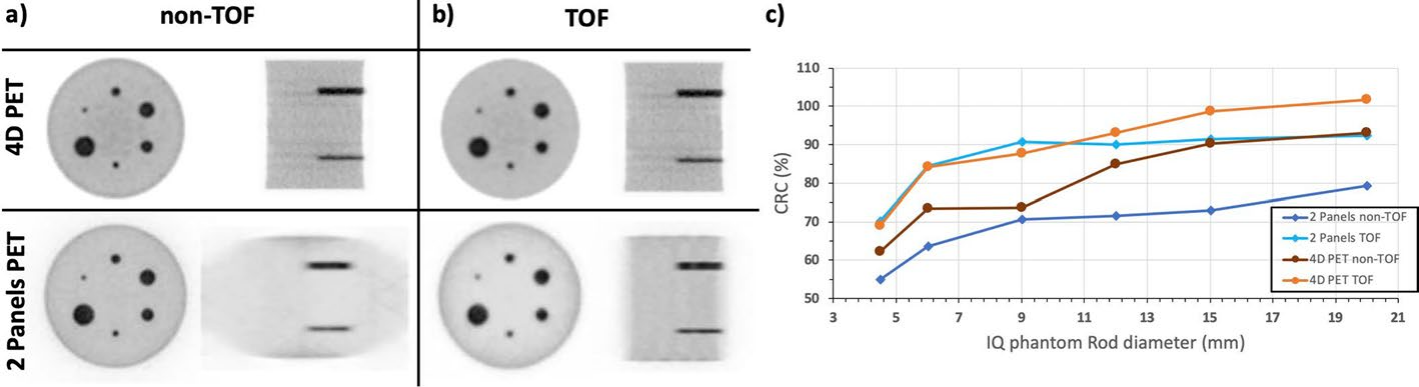
**a** and **b**, reconstructed images of the IQ phantom without and with including TOF capabilities in the 4D PET (top) and ODC (bottom) simulations, respectively. **c** CRC values of the IQ rods for each case

Regarding the IQ phantom simulations, similar effects are observed. The panels (a) and (b) in Figure 10, show coronal and axial views of the reconstructed IQ phantom for the 4D PET (top) and for the 2-panel ODC system (bottom). As in the Derenzo reconstruction, both the non-TOF and TOF cases are reported and, as expected, the effect of including TOF information is more evident in the ODC case since the axial view of the IQ phantom improves until almost recovering its expected shape and image contrast. The plot in Figure 10c shows the calculated CRC values for each IQ rod and for each case. In the non-TOF cases the CRC values worsens (on average) a factor of 12% between the ODC and 4D PET, while in the TOF cases the average CRC worsening is only of 3%. Also, the average improvement in the CRC values between the non-TOF and TOF cases are of 10% and 20% for the 4D PET and the ODC PET, respectively thus highlighting the major impact derived of including TOF information in open PET geometries.

Figures 9 and 10, prove that it is possible to achieve comparable imaging performance with ODC systems than conventional ring-shaped scanners.

### B. detector performance

**Fig. 11:**
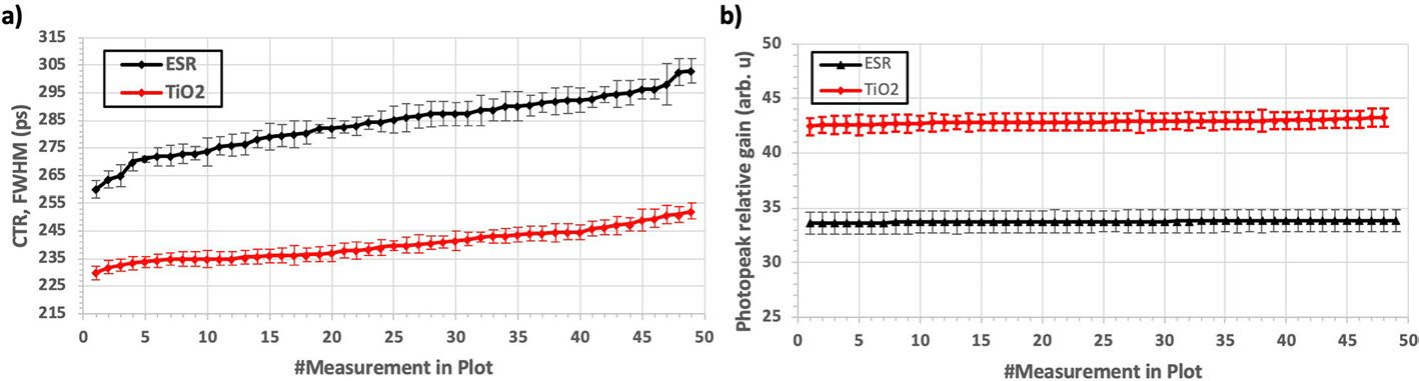
**a** measured CTR value for the 48 different configurations for both the ESR and TiO_2_ cases. **b** measured photopeak relative gain for the 48 different configurations for both the ESR and TiO_2_ cases

Figure 11a and b, shows the CTR and photopeak relative gain measured for the prototype detector element and for each evaluated treatment: ESR and TiO_2_ coatings. The results are presented in ascending order, from the best to the worst CTR performance. To understand the parameter configuration associated with each point, see Table 2.

Best CTR values of 260 $\pm$ 4 ps and 230 $\pm$ 2 ps were obtained for the ESR and TiO_2_ cases, respectively, after applying an energy filter of ± 30% at the photopeak (i. e., only those coincidence events falling within the range of 357 to 613 keV were considered for the CTR calculation). The parameter configuration yielding these results were (SiPM_OV_, Th_E_, Th_1_, Th_2_)_ESR_ = (5, 15, 23, 11) and (SiPM_OV_, Th_E_, Th_1_, Th_2_)_TiO2_ = (5, 15, 23, 17). Therefore, better performance in terms of CTR was provided by the TiO_2_ coating case.

Regarding photopeak relative gain, higher values (i.e., higher gain and thus light transferring to the photodetectors) were provided by the TiO_2_ coating case, with approximately a gain of 29% concerning the ESR case. The energy resolution, estimated as the ratio between the photopeak FWHM and the photopeak channel, was, on average, 11 $\pm$ 1% for the TiO_2_ case and degraded to 15 $\pm$ 1% for the ESR. It should be pointed out that the energy performance is less affected by the PETsys parameter configuration than the CTR.

Table 4 shows the best and average (overall measurement trials) CTR and maximum photopeak value achieved for each surface treatment. The last column depicts the improvement of these parameters when using TiO_2_ instead of ESR.

**Table 4:** Summary of the best and average measured CTR and photopeak energy channel

	ESR	TiO_2_	Improvement % (TiO_2_/ESR)
Best CTR (ps)	260 $\pm$ 4	230 $\pm$ 2	13.2
Average CTR (ps) *All measurements	284 $\pm$ 10	240 $\pm$ 6	17.9
Max. Photopeak Gain	34 $\pm$ 1	43 $\pm$ 1	28.5
Avg. Photopeak Gain * All measurements	34 $\pm$ 1	43 $\pm$ 1	27.0

The last column reports the improvement of these parameters when using TiO_2_ instead of ESR

The better CTR performance provided by the TiO_2_ can be explained by observing the reflectance spectra of the ESR and TiO_2_ since enhanced reflectivity for longer wavelength light emission (i.e., LYSO ∼ 360–600 nm, peaking at around 402 nm) [71] is provided by the white TiO_2_ coat (EJ-510) [45], see Table 5.

**Table 5:** Reflectance values for the selected materials at different wavelengths

	Reflectance (%)			
	310 nm	340 nm	370 nm	> 410 nm
ESR	23	9	35	93
TiO_2_	–	60	70	95

**Fig. 12:**
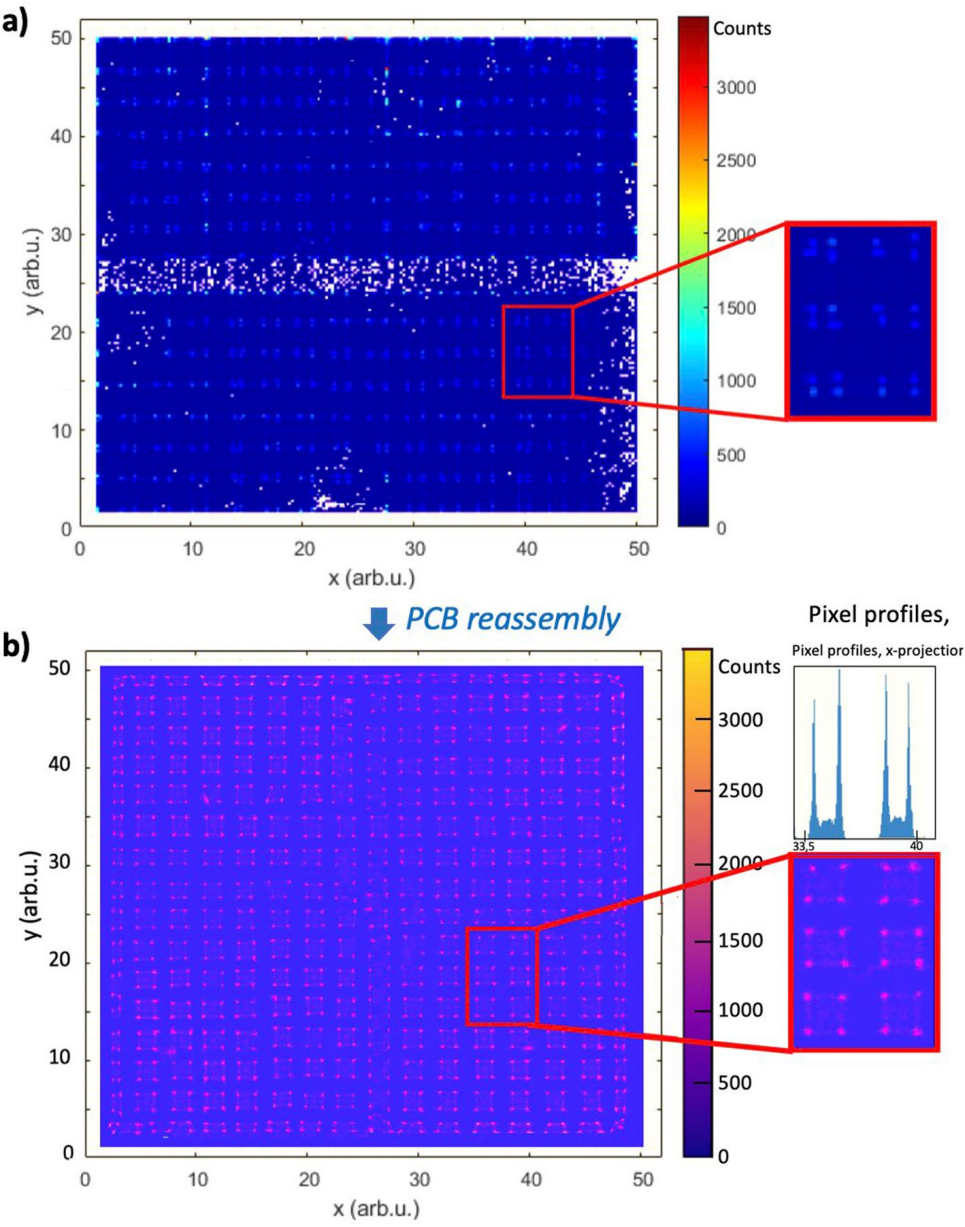
Flood maps of the entire module before **a** and after **b** PCB reassembly. Data was measured in coincidences. A zoomed area is shown to better visualize clusters of 4 pixels (couple to 1 SiPM)

Figure 12 panel (a) shows the acquired flood map using the parameter combination obtained in the previous experiment for the TiO_2_ case. In the first try, some PCB channels were missing, and thus, gaps and other effects were observed in the flood map. After reworking the PCB board, all channels were working and the flood map quality improved as can be see panel (b) of Fig. 12, the flood map demonstrates that all the 2 × 2x12 mm^3^ pixels are resolved. Indeed, looking at the zoomed area, one can see that the 4 pixels contained in one SiPM are clearly resolved. Additionally, the x-projection profiles of these pixels are provided to further demonstrate the spatial capabilities of the module. This proves that the designed module achieves spatial resolution values < 2 mm (pixel size). It should be mentioned that the two pixels closer to the edges are slightly compressed.

Before reworking the channels, CTR values of 243, 243, 245, 246 and 251 ps FWHM were obtained for the ROIs (4 scintillation pixels each) at SiPMs (8,8), (10,9), (2,6), (14,14) and (1,1), respectively, after energy filtering. After reworking the PCB and implementing time-skew corrections, these CTR values improve to 236, 233, 241, 243, 241, 246 for the same ROIs. The module CTR resolution, estimated as the mean value for the 5 ROIs, is 239 $\pm$ 4 ps, which demonstrated the successful scaling-up process of the prototype detector to a module.

## Discussion

The present work provides a detailed description of a proof of concept PET detector module appropriate for developing the new generation of ODC PET imagers suitable for guiding biopsy and clinical interventions during scanning.

The idea of constructing a two-panel system is not new and has been already proposed in other works such as in (Zeng, et al.) [66] or in (Li, et al.) [33], among others. It is also reported on the performance of a two-panel PET insert in (Razdevšek, et al.) [67], were the authors show the simulated performance of a PET consisting of two/four fast-timing flat-panels, or in (Peng) [68] were a simulation study of a cardiac-dedicated PET system based on a dual-panel geometry, among others. Moreover, it has been published a preliminary work in which the authors have extended such a two-panel concept for the construction of a “walk-through” total-body PET, named (WT-PET), as explained in (Vandenberghe, S., et al.) [32].

These previous works demonstrate the interest of the community for such a device. Our ODC proposal will provide a high-performance scanner with accurate TOF capabilities which will allow to guide biopsy and also, clinical interventions during scanning. Moreover, the variable FOV will allow to boost sensitivity, adapt to different patient sizes and enable new studies.

In particular, the proposed ODC panels cover an area of 256 × 256 mm^2^ each and allows for variable distances between them. Each panel is constructed using 4 × 4 modules, being each module formed by LYSO scintillator pixel arrays coupled to matrixes of SiPMs in a 4:1 configuration. Then, the TOFPET2 ASIC from PETsys is used to individually read each SiPM. For the second generation of this ODC PET system, we plan to implement a reduction readout system and achieve a signal compression ratio of 4:1 [41, 50], which is already under investigation in our group.

The expected performance of the proposed system has been evaluated through Monte Carlo simulations with GATE v9.2. The study includes the evaluation of the system sensitivity for three different distances between panels of 200, 250, and 300 mm since these are optimal values for imaging pediatric- [70], average- and plus-size-patients. Regarding image quality, simulations of the Derenzo phantom and the IQ NEMA NU-2 2008 phantom were performed. These phantom simulations were repeated but using a conventional full-ring scanner [54] to demonstrate the comparable performance of the ODC system with current state-of-the-art PET after including TOF capabilities. An MLEM algorithm was implemented for image reconstruction, which already includes TOF capabilities.

As shown in Fig. 5 and Table 3, the sensitivity worsens towards the edges of the scanners and with the distance between panels. Nevertheless, the achieved values are within the expected range and are suitable for a high-performance ODC-PET imager. For instance, the sensitivity values for distances between panels of 250 and 300 mm remains > 1% for a 10 cm range ($\pm$ 5 cm from the FOV center) which is large enough to cover organs like the prostate or the heart (the most likely organs to benefit from the proposed scanner). Regarding the shape of the sensitivity profile, these are in agreement with the ones reported by commercial scanners such as the Biograph mCT TOF PET/CT scanner (Siemens Molecular Imaging) which reports sensitivity values of 0.96% and 0.94% respectively at 0 cm and 10 cm from the center of the FOV (218 mm axial coverage) or, the Philips ingenuity TF PET/MR that reports similar values and a similar drop in sensitivity closer to the FOV edges. Therefore, our proposed ODC system is within the performance of clinical scanners [54].

We would like to mention that, using thicker scintillation crystals in the detectors is preferable since allow to boost sensitivity. However, using thick crystals also increases the spatial resolution dependency with the photon DOI in the scintillator and, if not accounted during image reconstruction, the final images suffers from blurring effects (known as parallax error), especially at the edges of the FOV. Therefore, to provide uniform spatial resolution across the entire FOV, DOI corrections are required. Furthermore, DOI corrections are more necessary for small aperture scanners such as preclinical PET and dedicated PET, like the proposed ODC one, since more oblique LORs are expected. Yet, providing DOI information in pixelated-based PET detectors is not straight forward and requires the implementation of complex methods or additional components (more SiPMs, staggered detectors, etc. [74]).

Aiming at minimizing parallax errors in the present detector design, we decided to use 12 mm long pixels since we are not providing DOI information at the moment. As part of future studies, we will investigate the implementation of a DOI encoding algorithm (we are currently trying DOI averaging methods with different number of neighboring pixels and light-sharing between pixels, among others), for the developed ODC detectors, and then, in a future design consider increasing the pixel length.

Regarding image quality, Figs. 6 and 8 show the reconstructed images of the IQ and Derenzo phantoms, respectively. In both cases, the reconstructed images present superior quality when TOF information is included since it allows for the mitigation of the artifacts produced by the lack of angular coverage (open geometries) [60]. The two smallest rods (1 and 1.2 mm) in the Derenzo phantom are not resolved, but the rest (1.6, 2.4, 3.2, and 4 mm) are clearly distinguished; thus, the expected image resolution is within the 1.6–2 mm range. Additionally, the comparative study of the same phantom images but simulated in a conventional full-ring brain dedicated PET shows similar imaging capabilities thus, these results justify the claim that enabling TOF capabilities compensates the lack of angular projections in ODC scanners.

Based on the simulation results, a prototype detector element was constructed. First, a single prototype detector element was built and two different pixel surface treatments (ESR and TiO_2_ coats) were evaluated. Best CTR and photopeak relative gain of 230 $\pm$ 2 ps and 43 $\pm$ 1 Ch, respectively, were achieved for the TiO_2_ case. These values show a CTR improvement of approximately 13.2% concerning the ESR. These results agree with similar studies published by other groups [17, 38–40]. Consequently, TiO_2_ coating was selected as a reflective material for the scaling-up process based on the detector prototype result. It should be mentioned that both the CTR performance and pixel resolvability in the flood maps were better at lower SiPM OV values, which seems contradictory with the fact that the photodetection efficiency (PDE) of the SiPM improves with OV and thus, the achievable CTR [48].We think that the better response at low OV is due to the combination of the different elements that compose the present detector and not only to the SiPM response by itself. For instance, we are being benefited by the LYSO high light yield and the TiO_2_ coating which has demonstrated to boost light collection efficiency in the SiPM since more optical photons are redirected towards the photosensor sensitive area and thus, generates high amplitude signals. Since the amplitude of the signals, as well as the total correlated noise and the dark noise, increases with the SiPM OV, the detectors may saturate earlier than expected, resulting in lower acquisition rates, worse signal characterization (worse CTR) and lower pixel resolvability (since with the implemented positioning algorithm most of the events are positioned towards the center of the SiPM), which may explain the better results at lower OV.

Moreover, we are using a custom designed PCBs for the SiPM—ASIC connections, which may introduce additional noise that increase at higher OV. Thus, the noise in our experimental setup may become more pronounced for higher OV, and thus the CTRs worse.

Then, an entire module composed of a matrix of 4 × 4 prototype detectors was built, and the preliminary results are reported in Fig. 12. As observed in the flood map, the clusters of 4 pixels are resolved (worse resolvability of the ones closer to the edges in which, due to compression effects, one column/row of pixels is compressed [72]). This result confirms that the spatial resolution of the modules is < 2 mm (pixel size), as required for the PoC. Note that this is a preliminary result based on home-made ensembled electronics, and some ASIC channels were lost in the first validation but, after reworking these channels, the gaps vanished. However, the flood image is still suffering from compression effects. We believe that when having the final PCBs the results will further improve.

Regarding TOF capabilities, an average CTR resolution (obtained as the average for 5 ROIs of 16 pixels each) of 239 $\pm$ 4 ps was measured, thus demonstrating the successful scaling-up process. Note that the reported CTR value accounts for the possible differences in the temporal response between the two coincidence modules which is affected by the tracer length in the PCB design, level of intrinsic electronical noise, impedance mismatches between the different parts, speed in response to an event and signal transfer, inhomogeneities in the scintillator-photosensors coupling, or misalignments between the modules and the source, to mention but a few. Yet, in the ideal scenario in which the two coincidence modules are identical these differences will be minimize and thus their contribution to the global CTR. Considering this, we believe the CTR value could improve to meet our targeted 200 ps since, in addition to the previous factors, in these measurements, the PCB was reworked by ourselves, and only time-skew correction [73] have been applied but not time-walk corrections. We are investigating the best way to include this correction which accounts for the difference between the time signals of the channels due to different electronic paths. Moreover, we detected some other limitations in our experiments, mostly related to: (i) the mechanical components: holders, shielding boxes, stage-motors and source alignment (which is crucial to achieve good CTR values); (ii) the temperature, which played an important role and, although we are using high efficiency cooling set-ups based on compressed air and Vortex tubes, some fluctuations may happen (since the acquisition of all SiPM and ASIC settings lasted several days) thus affecting the experimental results; and (iii) slight misalignments between pixels in the scintillation matrix have been detected as well as worse uniformity of the TiO_2_ coating in some areas. For future test we will address these factors.

Summarizing, the present manuscript reports the design and experimental validation of a PET module suitable for constructing a 2-panel PET system that may be the key to unlock the new generation of ODC PET scanners; the obtained results are already optimal for our goal, and improvements are expected when including the corrections mentioned above.

In the following months we will produce 32 more detector modules. These modules will be mounted in two panels and the NEMA 2008 protocol will be studied. Both the simulation and experimental results suggest that this scanner is a potential candidate for the new generation of ODC PET scanners which may ease the way for real-time functional/metabolic molecular image-guided interventions in oncology, such as breast, or prostate biopsy, focal ultrasound or radiation therapy, in cardiology and neurology.

## Conclusion

The simulation study of the proposed two panel PET scanner showed a spatial resolution of $\sim$ 1.6–2 mm and a uniformity value of around 87%. Furthermore, we have demonstrated that TOF information minimizes the image artifacts produced by the lack of angular projections through a comparative study with the same phantom simulations but performed in a conventional dedicated ring-PET in which all angular views are covered.

For the construction of the detector module, experiments showed the best performance when using TiO_2_ coating (achieving 15% better than its closest competitor, ESR on 5 polish surfaces) as treatment for the scintillation pixels. An experimental CTR value of 239 $\pm$ 3 ps was obtained for a full module (1024 LYSO pixels, 256 SiPMs and 4 ASICs) and the flood map image suggests that all pixel elements (showing compression in the ones closer to the edges) are resolved thus a spatial resolution below the pixel size (2 mm) is reached.

## Data Availability

The data supporting the results of this study are available upon reasonable request to the corresponding author.

## Abbreviations

CRC: Contrast recovery coefficients
CTR: Coincidence time resolution
DAQ: Data acquisition system
ESR: Enhanced Specular Reflector
FOV: Field-of-View
FWHM: Full-Width-At-Half-Maximum
IQ: Image quality
LOR: Line of response
MLEM: Maximum Likelihood Expectation Maximization
ODC: Open Detector Configurations
OV: Over voltage
PCB: Printed Circuit Board
PDE: Photon detection efficiency
PET: Positron Emission Tomography
PoC: Proof of concept
ROI: Region of interest
RSDC: Ring-Shaped Detector Configuration
SiPM: Silicon Photomultiplier
SNR: Signal-to-Noise ratio
TOF: Time-of-Flight
WB-PET: Whole-body (WB-) PET
WT-PET: Walk-through (WT-) PET
